# Preimplantation Genetic Diagnosis of Neurodegenerative Diseases: Review of Methodologies and Report of Our Experience as a Regional Reference Laboratory

**DOI:** 10.3390/diagnostics9020044

**Published:** 2019-04-23

**Authors:** Chun-Hua Liao, Ming-Yuh Chang, Gwo-Chin Ma, Shun-Ping Chang, Chi-Fang Lin, Wen-Hsiang Lin, Hsin-Fu Chen, Shee-Uan Chen, Yi-Chung Lee, Chi-Chao Chao, Ming Chen, Sung-Tsang Hsieh

**Affiliations:** 1 Department of Pediatrics, National Taiwan University Children’s Hospital, Taipei 10041, Taiwan; shiva90345@gmail.com; 2 Division of Pediatric Neurology, Department of Pediatrics, Changhua Christian Children’s Hospital, Changhua 50050, Taiwan; 54090@cch.org.tw; 3 Department of Genomic Medicine and Center for Medical Genetics, Changhua Christian Hospital, Changhua 50046, Taiwan; 128729@cch.org.tw (G.-C.M.); 70914@cch.org.tw (S.-P.C.); 397620cch@gmail.com (W.-H.L.); 4 Department of Genomic Science and Technology, Changhua Christian Hospital Healthcare System, Changhua Christian Hospital, Changhua 50046, Taiwan; 5 Department of Medical Laboratory Science and Biotechnology, Central Taiwan University of Science and Technology, Taichung 40601, Taiwan; 6 Department of Obstetrics and Gynecology, College of Medicine and Hospital, National Taiwan University, Taipei 10041, Taiwan; enokialin@gmail.com (C.-F.L.); hfchen@ntu.edu.tw (H.-F.C.); csu@ntu.edu.tw (S.-U.C.); 7 Graduate Institute of Medical Genomics and Proteomics, College of Medicine, National Taiwan University, Taipei 10051, Taiwan; 8 Department of Neurology, Taipei Veterans General Hospital, Taipei 11217, Taiwan; ycli@vghtpe.gov.tw; 9 Department of Neurology, National Taiwan University Hospital, Taipei 10048, Taiwan; shsieh@ntu.edu.tw; 10 Department of Life Science, Tunghai University, Taichung 40704, Taiwan; 11 Department of Molecular Biotechnology, Da-Yeh University, Changhua 51591, Taiwan; 12 Department of Anatomy and Cell Biology, College of Medicine, National Taiwan University, Taipei 10051, Taiwan; 13 Graduate Institute of Brain and Mind Sciences, College of Medicine, National Taiwan University, Taipei 10051, Taiwan

**Keywords:** PGD, ARMS-qPCR, FAP, spinocerebellar ataxia, Huntington’s disease, linkage marker

## Abstract

Preimplantation genetic diagnosis (PGD) has become a crucial approach in helping carriers of inherited disorders to give birth to healthy offspring. In this study, we review PGD methodologies and explore the use of amplification refractory mutation system quantitative polymerase chain reaction (ARMS-qPCR) and/or linkage analysis for PGD in neurodegenerative diseases that are clinically relevant with typical features, such as late onset, and which are severely debilitating. A total of 13 oocyte retrieval cycles were conducted in 10 cases with various neurodegenerative diseases. Among the 59 embryos analyzed, 49.2% (29/59) were unaffected and 50.8% (30/59) were affected. Of the 12 embryo transfer cycles, three resulted in pregnancy, and all pregnancies were delivered. The implantation rate and livebirth rate were 23.1% (3/13) per oocyte retrieval cycle and 25.0% (3/12) per embryo transfer cycle. Allele dropout (ADO) was noted in two embryos that were classified as unaffected by ARMS-qPCR but were evidenced as affected after prenatal diagnosis, rendering the false negative rate as 6.3% (2/32). Four among the 13 cycles underwent PGD by ARMS-qPCR coupled with linkage analysis, and all were correctly diagnosed. We conclude that PGD by ARMS-qPCR and/or linkage analysis is a feasible strategy, whereas ADO is a concern when ARMS-qPCR is used as the sole technology in PGD, especially in autosomal dominant diseases.

## 1. Introduction

### 1.1. Overview of Preimplantation Genetic Diagnosis

Preimplantation genetic diagnosis (PGD), invented in 1990, has become the standard of care for halting the transmission of inheritable diseases to the next generation [1,2]. Polymerase chain reaction (PCR)-based technology is one of the major molecular technologies used in PGD. Direct mutation detection, such as Sanger sequencing and amplification refractory mutation system quantitative PCR (ARMS-qPCR) complemented by linkage analysis of the co-amplification of polymorphic short tandem repeat (STR) markers, are being widely adopted [3,4,5] because both linkage analysis and direct mutation detection assays have shortcomings. The direct mutation detection assay can be used in a three-day cleavage stage biopsy when fresh embryo transfer is desired and can be coupled with frozen embryos if a five- or six-day blastocyst stage biopsy is chosen. However, in selected urgent cases, a timely overnight diagnosis is still feasible. Such timely genotyping is prone to allele dropout (ADO) and further confirmation by repeat experiments or by additional genotyping methods is impossible. ADO can result in catastrophic misdiagnosis in PGD, especially in autosomal dominant (AD) disorders [4,6,7]. Linkage analysis cannot detect recombination within the segment separating the linked polymorphic markers and the disease loci. Combining more informative linkage markers is advisable to reduce the chance of misdiagnosis. The genotyping methodologies used in PGD are summarized in Table 1.

### 1.2. Current Status of PGD in Neurodegenerative Diseases

Neurodegenerative diseases are a group of disorders characterized by the progressive decline of neurological function due to neuronal degeneration in the central and peripheral nervous systems. They have no curative therapies. The diseases are further categorized according to the involvement of the nervous system. For example, Huntington’s disease and spinocerebellar ataxia (SCA) mainly affect the central nervous system, while whereas Charcot–Marie–Tooth disease mainly involves the peripheral nervous system. Based on the mutation type, neurodegenerative diseases can be categorized into (1) triplet repeat expansion diseases (e.g., Huntington’s disease, SCA, fragile X syndrome, and myotonic dystrophy), (2) aberrant gene dosage or rearrangement diseases (e.g., Charcot–Marie–Tooth type 1A and spinal muscular atrophy (SMA)), and (3) point mutation or small insertion/deletion diseases (e.g., familial amyloidotic polyneuropathy (FAP)). PGD has been applied to those diagnosed with a variety of neurodegenerative diseases to halt their transmission to the next generation.

To understand the status of PGD application in neurodegenerative diseases, a systemic survey was administered by retrieving relevant literature from PubMed through March 2019. The searching strategies and keywords included “preimplantation genetic diagnosis”, “PGD”, “Huntington”, “neurodegenerative”, “spinocerebellar ataxia”, “spinal muscular atrophy”, “SMA”, “prion”, “familial amyloidosis”, “Charcot–Marie–Tooth”, and “CMT”. An initial search identified 92 articles. As we focused on the application of PGD in neurodegenerative disorders (with an emphasis on molecular techniques), studies exclusively concerning ethical issues, diagnostic technique evolution, or those that included patients with non-neurodegenerative diseases were excluded. A total of 23 original articles fulfilled these criteria, and their major findings are summarized in Table 2.

A large proportion of studies focused on Huntington’s disease, which is an AD neurodegenerative disease characterized by (1) a late onset and progressive course and (2) symptoms such as chorea, emotional problems, and cognitive decline [32]. Treatment is supportive and centered on alleviating abnormal movements and psychiatric symptoms. The mean age of onset is 35 to 44 years with a median survival of 15–18 years [33,34]. Accordingly, Huntington’s disease is often diagnosed when the patient’s children approach reproductive age. Due to a lack of effective treatments and high genetic penetrance, individuals at risk of disease often face dilemmas; for example, deciding whether to undergo pre-symptomatic diagnosis, and whether to marry or have biological offspring. PGD has become a feasible solution for at-risk individuals wishing to have unaffected biological offspring. Another disease included in our survey is FAP. FAP is a devastating neurodegenerative disease that damages the motor, sensory, and autonomic components of the peripheral nerves and has an AD transmission mode. Mutations in various amyloidogenic proteins underlie FAP, with the transthyretin gene (*TTR*; MIM #176300) being the most frequently mutated [35,36,37,38]. *TTR*-related FAP (*TTR*-FAP; MIM #105210) has different clinical manifestations and is mainly classified as neuropathic, oculoleptomeningeal, and cardiac disease by various phenotypes [38].

Worldwide, more than 100 mutations in *TTR* have been identified [39], with the c.148G>A (p.V50M) (legacy V30M) mutation being the most common. Other mutation hotspots of *TTR* were reported in various ethnic groups; in particular, the c.349G>T (p.A117S) (legacy A97S) mutation was reported in Taiwanese FAP patients [40]. In contrast to the more common c.148G>A (p.V50M) variant with an onset age of ca. 35 years old, the neurological deficits of c.349G>T (p.A117S) start at around 60 years of age. The disease course of c.349G>T (p.A117S) was apparently more rapid than that of c.148G>A (p.V50M), with patients becoming bedridden in eight years [41]. Children of patients with c.349G>T (p.A117S) were at reproductive age when their parents became symptomatic. These at-risk carriers have an urgent need for genetic counseling.

## 2. Materials and Methods 

### Genotyping Strategies Adopted in Our Laboratory

**Figure 1:**
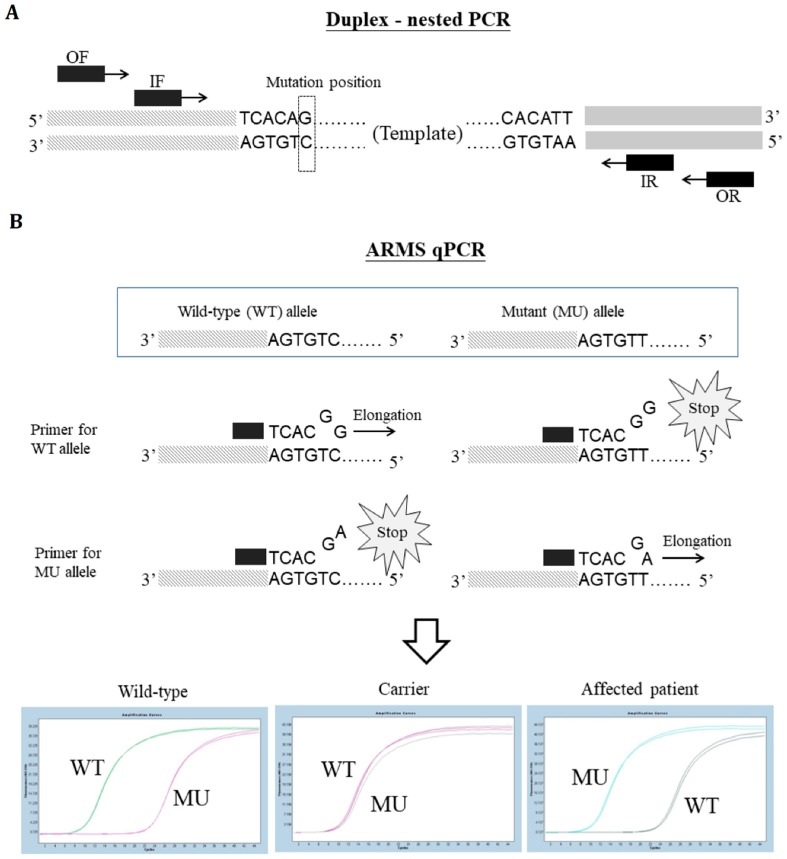
Schematic diagram of a duplex-nested amplification refractory mutation system quantitative polymerase chain reaction (ARMS-qPCR) for the preimplantation genetic diagnosis (PGD) of a point mutation. (**A**) Duplex-nested PCR was used to amplify a region including the mutation position. OF and OR, outer primer set; IF and IR, inner primer set. (**B**) ARMS-qPCR using different primers to separate the wild-type (WT) and mutant (MU) alleles. A representative ARMS-qPCR experiment for the following genotypes in duplication: wild-type (homozygous WT), carrier (heterozygous WT/MU), and affected patient (homozygous MU). Black and grey dotted histograms indicate the primers and template deoxyribonucleic acid (DNA). Different color curves in Figure 1B indicate different runs of ARMS-qPCR testing.

We devised a novel in-house patented ARMS-qPCR genotyping strategy (Figure 1) to address the need for timely and overnight diagnosis of fresh embryo transfer when a trophectoderm biopsy was performed at the 5/6-day blastocyst stage, since our protocol only includes 5/6-day blastocyst biopsies. PGD by ARMS-qPCR is cost-efficient and time-saving, especially compared to next-generation sequencing-based methods [42] or linkage analysis, which involve experimentally screening multiple linked markers to select the informative ones [4]. However, given the concern with ADO, which renders a false negative diagnosis as we describe later in the results section, we adopted a strategy since 2016 that, in AD disorders where point mutation is the mutation type, at least two of the following methodologies need to be applied: Sanger sequencing, linkage analysis with STR markers, and ARMS-qPCR. Whether fresh embryo transfer can be achieved depends upon the individual situation since the time spent on each PGD case may vary. For trinucleotide expansion disorders, such as the poly-Q diseases, the nested PCR amplification of the causative loci followed by capillary electrophoresis to determine the lengths of the alleles remains our main strategy. All embryos that were classified as inappropriate for transfer need to be confirmed before being discarded. All pregnant cases need to receive confirmatory invasive prenatal diagnosis to avoid the livebirth of affected babies. Lastly, the preclinical setup of the PGD by ARMS-qPCR is routinely performed before couples seeking the assistance of PGD are enrolled in the clinical PGD services. Documents of diagnostic examination informed consent were obtained from all couples subjected to the clinical preimplantation genetic studies. This retrospective study is a chart review and the request for the waiver of informed consent was approved by the Research Ethics Committee of National Taiwan University Hospital, Taiwan (REC No.: 201510127RIND; approval date: 26 Jan 2016).

## 3. Results

### 3.1. Overall Outcomes

A total of 10 patients with various neurodegenerative disorders underwent 13 oocyte retrieval cycles for in vitro fertilization (IVF) with PGD at the core laboratory of Dr. Ming Chen, a major PGD laboratory in Taiwan, during 2013–2016 (Table 3). The indications included SCA type 3 (SCA3) and SCA6, Charcot–Marie–Tooth disease type 2E, Huntington’s disease, amyotrophic lateral sclerosis (ALS), FAP, and SMA. A total of 59 embryos were sent for PGD and the successful diagnosis rate was 100% (59/59). The involved genotyping methodology included linkage analysis for those with trinucleotide expansion disorders (SCA3, SCA6, and Huntington’s disease) in 21 embryos from 4 patients, and ARMS-qPCR method for those with point mutations (Charcot–Marie–Tooth 2E, ALS, and FAP) and small deletions (SMA) (although SMA is a gene dosage disorder, SMN2 has a small deletion in exon 7 compared to SMN1 that can be used in PGD to elucidate the existence of SMN1 and thus exclude the affected embryos) in 38 embryos from 6 patients. All the embryos classified as affected were confirmed before being discarded, and therefore the false positive rate was 0%. Three patients delivered normal healthy babies (one singleton and two twins); the livebirth rate (take-home baby rate) was 23.1% (3/13) per oocyte retrieval cycle and 25.0% (3/12) per transfer cycle (Table 3). Apart from the cases reported in Table 3, other couples with diseases such as Charcot–Marie–Tooth disease type 1A had undergone counseling and planning for IVF with PGD but had not actually entered and completed the cycle, and thus were not enrolled. Notably, two embryos from two cases (Case 5 and Case 9 in Table 3) were misclassified as unaffected, and thus the false negative rate was 6.3% (2/32). The two false negative cases actually resulted in healthy babies after selective reduction was performed. Case 5 was described in our earlier publication [4] and Case 9 is described in detail in the following section. All the babies being born were confirmed to be unaffected by postnatal genotyping.

### 3.2. Example for ADO: The Index Family of FAP

Case 9 (Table 3) is a 29-year-old female asymptomatic FAP carrier who wished to have unaffected offspring. Her father, the index case, started to have numbness over the lateral side of his left thigh at the age of 50 years. The numbness progressed to his left calf, right leg, and bilateral upper extremities (with a pattern of extension from finger tips to the forearm), and was followed by bilateral hand weakness. Other past history was unremarkable.

A neurological examination showed involvement of the motor, sensory, and autonomic systems. Muscle strength was reduced symmetrically (4 to 5 according to the Medical Research Council scale) with generalized hyporeflexia in upper limbs and areflexia in lower limbs. Sensations to pinpricks and temperature stimuli distal to the bilateral thighs and forearms were impaired. Orthostatic hypotension, impotence, and marked diarrhea were present, indicating autonomic involvement.

**Figure 2:**
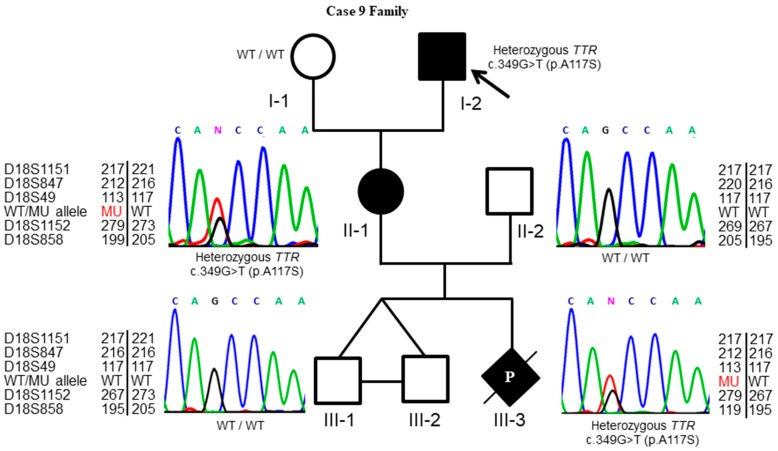
Pedigree and *TTR* genotyping of an index family with FAP. Case II-1 (i.e., Case 9 in Table 3), carrying a familial c.349G>T (p.A117S) mutation, adopted PGD and a triplet pregnancy was achieved after transferring two unaffected embryos. Prenatal diagnosis by direct sequencing and linkage analysis with give informative microsatellite markers (D18S1151, D18S847, D18S49, D18S1152, and D18S858) revealed unaffected identical twins (III-1 and III-2) and one affected singleton (III-3), indicating the occurrence of ADO in PGD. Filled and open symbols represent affected and unaffected individuals, respectively; circles, squares, and diamonds indicate females, males and individuals of unknown sex, respectively; P with a diagonal line indicates selective fetal reduction during the pregnancy. The arrow indicates the affected proband (I-2). C, A, G, N indicated the cytosine, adenine, guanine and ambiguous base calling respectively in the DNA sequence.

Nerve conduction studies showed reduced amplitudes of the sensory action potential and the compound muscle action potential with slowing of conduction velocities in most sampled nerves, compatible with polyneuropathy involving motor and sensory nerves. Cardiac autonomic function tests revealed reduced R-R interval variation at rest and during deep breathing. Given the multisystem involvement, genetic testing was conducted and demonstrated a c.349G>T (p.A117S) mutation in the *TTR* gene. His condition deteriorated rapidly and he became bed-ridden. Nasogastric tube feeding was initiated due to severe dysphagia. The patient’s daughter (Case 9) also tested positive for the c.349G>T (p.A117S) mutation. Due to her wish to conceive a healthy child, she was referred for PGD and IVF (Figure 2).

Trophectoderm cells obtained through a Day 6 blastocyst biopsy were collected at the National Taiwan University Hospital Reproductive Fertility Center. Primer sets for ARMS-PCR for PGD were designed and evaluated in the same manner as we previously reported [4,6]. The ARMS-PCR for PGD required two PCR steps. First, two primer sets of duplex-nested PCR (1F: 5’-TTTCCAGCTCCAGAATGCTAA-3’/1R: 5’-TGCTTGCAAGACAATGGAAA-3’; 2F: 5’-TGCAGCAGCTCTTCAATGAC-3’/2R: 5’-GCGTTCTGCCCAGATACTTT-3’) were used to amplify the region between the intron 3 and exon 4 of the *TTR* gene. Next, two sequence-specific forward primers (c.349G-wt: 5’-CTTCTCTCATAGGTGGTATTCACGG-3’ and c.349T-mu: 5’-CTTCTCTCATAGGTGGTATTCACGT-3’) were used to amplify wild-type and mutant alleles, respectively, with a 3’ mismatch at the penultimate nucleotide position to increase specificity. The same reverse primer (3R: 5’-ATTCCTTGGGATTGGTGACG-3’) was used for amplifications of both alleles. The wild-type and mutant alleles were distinguished by assessing the threshold cycle (Ct) value through the qPCR on a LightCycler 480 system (Roche, Basel, Switzerland). The amplified fragments obtained from the nested PCR were also subjected to bidirectional sequencing with the Big-Dye Terminator v3.1 Cycle Sequencing Kit and ABI Prism 3100 genetic analyzer (Applied Biosystems, Foster City, CA, USA) for confirmation.

During the PGD, six embryos with good morphologies were selected and biopsied from blastocysts on Day 6. Trophectoderm cells were examined by ARMS-qPCR to detect the presence of the familial c.349G>T (p.A117S) mutation and confirmed by Sanger sequencing. Two embryos were selected for transfer since they were unaffected by the mutation. The couple underwent IVF successfully, resulting in a triplet with one monozygotic twin pregnancy. Chorionic villus sampling was performed at a gestational age of 13 weeks, and direct sequencing coupled with linkage analysis revealed unaffected identical twins and one affected singleton, indicating the occurrence of ADO (Figure 2). After nondirective genetic counseling, the couple opted for selective fetal reduction. We applied linkage analyses and sequencing to ensure the correct fetus would be reduced, and the entire procedure was uneventful. Identical twin boy babies without the c.349G>T (p.A117S) mutation were delivered via cesarean section at 37 weeks of gestation age. The babies’ birth weights were 2496 g and 2824 g.

This index family illustrated the role of PGD in the delivery of healthy babies by an FAP-carrier mother. Notably, concerns persist about long-term neurocognitive outcomes for children conceived after PGD. Children born after PGD were evaluated at five years of age and showed normal developmental neuropsychological outcomes [43]. In contrast, preimplantation genetic screening (PGS) was found to be associated with inferior neurodevelopment in twins, but no specific difference was noted in singletons [44]. As these studies were limited by sample size, a larger longitudinal study is warranted to assess the physical, neurological, and cognitive development of children conceived after PGD. 

The ADO noted in Case 9 highlighted a risk of PGD that is especially concerning in AD disorders: an ADO will misclassify the affected embryo as unaffected, which can result in the birth of affected offspring. Invasive prenatal diagnosis is indispensable with regards to avoiding such complications, and selective fetal reduction is a feasible tool to correct the error [4]. The ARMS-qPCR used in PGD is therefore particularly more suitable as a supplement to the gold standard of linkage analysis than as a stand-alone procedure. It is especially helpful when informative markers are not easily found, and can minimize the risk of false negative results caused by recombination events between the selected informative markers and the disease locus [4].

## 4. Discussion

### 4.1. ADO in Our FAP PGD Case and Clinical Relevance

Only a limited number of reports have documented the use of PGD in FAP despite FAP being endemic in regions of Portugal, Sweden, Japan, and Brazil [45,46]. In Portugal, the prevalence of FAP is 0.41 per 2000 inhabitants and greater than 1 per 2000 inhabitants in 19 municipalities [47]. Owing to the relatively high prevalence and predictable disabling disease course, the use of PGD by FAP patients in Portugal appears reasonable to prevent the transmission of affected genes. However, the use rate of PGD in Portugal was only around 20.7% according to a self-administered questionnaire performed between January and May 2013. This was probably related to socioeconomic status, as a household income above €1000 per month is directly associated with the use of PGD [48]. 

The children of late-onset FAP patients are of reproductive age. In this context, PGD has been proven over the years to be a valid reproductive option for couples at risk of a specific genetic disease who wish to have unaffected offspring, preventing the traumatic termination of an affected pregnancy [5,49,50]. Numerous techniques, including a single-cell PGD use for the detection of the c.148G>A (p.V50M) mutation in the *TTR* gene [29], have been developed and applied to performing PGD for many rare genetic disorders, and the number of cycles increases each year [4,51,52]. In the PGD in Case 9, ARMS-qPCR was applied and twin babies free from the c.349G>T (p.A117S) mutation in the *TTR* gene were successfully delivered. This provides a foundation for the PGD of other neurodegenerative diseases. Notably, ADO is always a serious concern in the ARMS-qPCR strategy [4]. Differences in primer annealing efficiency, causing the preferential PCR amplification of one allele relative to another, may account for the occurrence of ADO. When using ARMS-qPCR in autosomal recessive (AR) disorders, two ADO events are necessary to cause a false negative. However, one ADO event can cause a false negative in AD disorders. As a result, ARMS-qPCR should be used with caution in AR disorders and is not suitable as the sole PGD technique in AD disorders.

### 4.2. Ethical Aspects 

There are controversial ethical aspects about the use of PGD in late-onset neurodegenerative diseases, similar to concerns raised in pre-symptomatic testing scenarios. As an example, for a couple receiving PGD without prior knowledge of their own carrier status, the presence of a mutant allele would be revealed if any affected embryos were found. If the couple chose not to be informed of the PGD results but requested that only unaffected embryos be transferred, then a scenario where no available embryos can be transferred would cause difficulties, or where the couple do not actually carry the mutant allele but a non-disclosure PGD cycle still needs to be conducted even if the biopsy is not needed or not conducted. In addition, medical teams would be under considerable stress, and it is often impractical to expect complete confidentiality in a real-world setting [53]. In our series, all couples chose PGD only after they decided to know their status, were confirmed to carry the mutant allele, and had received detailed, cautious, and non-directive genetic counseling. Notably, exclusion protocols (that is, not transferring all embryos with the same haplotype as the affected parent) should be considered in some cases. They have the advantage that the affected parent (usually with AD or late-onset neurodegenerative disorders such as Huntington’s disease) can choose to not know their genotype status, whereas a disadvantage is that the number of transferable embryos will be apparently reduced, inevitably affecting the fertility outcome (such as the implantation rate and livebirth rate) [54].

### 4.3. Concurrent PGD/PGS 

Outcome indicators of PGD include a successful diagnosis rate (the number of embryos for which diagnosis was provided divided by the total number of embryos being biopsied), implantation rate (the number of embryos implanted divided by the total number of embryos being transferred), and livebirth rate (the rate of liveborn pregnancy per transferred cycle or the rate of liveborn pregnancy per oocyte-retrieval) [3,4,5,6,7]. Whether frozen embryo transfer or fresh embryo transfer provides superior livebirth rates is still controversial. Nonetheless, it is critical for PGD laboratories to develop a genotyping platform compatible with the strict time limits required in fresh embryo transfers, especially the overnight turnaround time for a five or six-day blastocyst biopsy. With an increase in the popularity of PGS, there is a growing need for concurrent PGD/PGS. Currently, the strategies used in PGS include array-based (either array-comparative genomic hybridization or single nucleotide polymorphism chromosomal microarray) techniques [55,56,57], qPCR-based techniques [58,59], and next-generation sequencing (NGS)-based techniques [26,42,60,61]; we are excluding the outdated fluorescence in situ hybridization (FISH)-based diagnostics [62]. Some of the techniques were reported to be successfully applied in concurrent PGD/PGS [21,26,63,64]. Some women will inevitably opt for PGD to select embryos unaffected by certain heritable monogenic disorders as well as to select euploid embryos, which will reduce the chance of abortion due to aneuploidy in the later gestational period or improve the implantation rate [65]. However, we recommend that concurrent PGD/PGS should be offered to all women undergoing PGD after more randomized trials have convincingly proven the efficacy of PGS [65]. More trials are needed given recent reports that show euploid babies born after the transfer of mosaic aneuploid embryos into the womb [66] and that question the consistency of PGS across laboratories adopting different genotyping technologies [67,68,69,70]. Couples who opt for concurrent PGD/PGS should be counseled that additional selection (namely, PGS) reduces the number of embryos being classified as appropriate for transfer and hence reduces the outcome indicators of PGD, among which the livebirth rate is included [8].

## 5. Conclusions

The rapid advances in PGD technology have enabled the prevention of the transmission of neurodegenerative diseases to future generations. PGD by ARMS-qPCR is a rapid, feasible strategy for such complicated diseases. However, considering the possibility of ADO during the use of the PCR-based method, ARMS-qPCR should be performed with caution in AR disorders and is not suitable as the sole PGD technique in AD disorders. For the PGD of neurodegenerative diseases, robust methodologies, proper genetic counseling covering technical and ethical aspects of genetic testing, and confirmatory invasive prenatal diagnosis are important.

## Figures and Tables

**Table 1:** Mutation types and the genotyping methodologies used in preimplantation genetic diagnosis (PGD).

Mutation Type	Disease (Gene) Examples	Genotyping Method	PGD Method
Inversion	Rare in neurodegenerative disorders	• I-PCR • Long-distance PCR • Southern blotting	• Linkage analysis
Point mutations	Common in neurodegenerative disorders, e.g., ALS (*FUS*), FAP (*TTR*)	• Direct DNA sequencing • DHPLC • TTGE	• ARMS-qPCR • Linkage analysis • Mini-sequencing
Small deletion/insertion (<1 exon)	Less common in neurodegenerative disorders, e.g., SMA (*SMN1*, *SMN2*)	• MLPA • Direct DNA sequencing	• ARMS-qPCR • Linkage analysis • Mini-sequencing • MARSALA
Large duplication (≥1 exon)	Charcot–Marie–Tooth 1A (*PMP22*)	• MLPA • High resolution aCGH	• Linkage analysis
Trinucleotide expansion	Very common in neurodegenerative disorders, e.g., Huntington’s disease (*HTT*), many subtypes of spinocerebellar ataxia (*ATXN1*, *MJD1/ATXN3/SCA3*), Poly Q diseases (*ATN1*, *AR*)	• Southern blotting • PCR followed by capillary electrophoresis (if CAG repeat number less than 100)	• Linkage analysis • Nested PCR followed by capillary electrophoresis (if CAG repeat number less than 100)

I-PCR = inverse polymerase chain reaction, MARSALA = mutated allele revealed by sequencing with aneuploidy and linkage analyses, ALS = amyotrophic lateral sclerosis, FAP = familial amyloidotic polyneuropathy, DHPLC = denaturing high performance liquid chromatography, TTGE = temporal temperature gradient gel electrophoresis, ARMS-qPCR = amplification refractory mutation system quantitative polymerase chain reaction, SMA = spinal muscular atrophy, MLPA = multiplex ligation-dependent probe amplification, aCGH = array comparative genomic hybridization. The table was modified based on Chen et al. [8].

**Table 2:** Methodologies used for the PGD of neurodegenerative disorders.

Disease	PGD Method	Strategy	Cycles	Pregnancy Rate Per Oocyte Retrieval	Pregnancy Rate Per Embryo Transfer	Notation	Reference
*Triplet repeat expansion*							
Huntington’s disease	Single-cell PCR	Direct diagnosis	9	1/153	1/13		Sermon et al. [9]
Huntington’s disease	Multiple-cell PCR	Direct diagnosis	15	NA	NA	Nondisclosure testing ^a^	Stern et al. [10]
Huntington’s disease	Single-cell PCR	Exclusion testing	7	1/67	1/6		Sermon et al. [11]
Huntington’s disease	Single-cell WGA followed by PCR	Direct diagnosis	1	1/15	1/2		Chow et al. [12]
Huntington’s disease (and P450 oxidoreductase deficiency)	Single-cell PCR or two-cell PCR	Direct diagnosis	2	0/18	0/2		Alberola et al. [13]
Huntington’s disease	Single-cell PCR	Direct diagnosis or exclusion testing	7	NA	2/10		Peciña et al. [14]
Huntington’s disease	Single-cell PCR	Direct diagnosis or exclusion testing	434 started; 389 continued to oocyte retrieval	105/5218	105/511		Van Rij et al. [15]
Huntington’s disease		Direct diagnosis and Linkage analysis	1	1/16	1/1		Perminov et al. [16]
SCA2	Single-cell PCR	Direct diagnosis	2	2/36	2/4		Moutou et al. [17]
SCA3	Single-cell PCR	Direct diagnosis	1	1/10	1/2		Drüsedau et al. [18]
Huntington’s disease (and myotonic dystrophy, fragile X syndrome)	Two-cell PCR	Direct diagnosis	NA	NA	NA	No implantation data	Sermon et al. [19]
*Gene dosage or rearrangement*							
Charcot–Marie–Tooth 1A	Single-cell PCR or two-cell PCR	Linkage analysis	2	1/18	1/2		Löfgren et al. [20]
Charcot–Marie–Tooth 1A	Two-cell PCR	Direct diagnosis	13	3/138	3/11		De Vos et al. [21]
Charcot–Marie–Tooth 1A	Single-cell PCR or two-cell PCR	Direct diagnosis	6	4/117	4/13		Lee et al. [22]
Charcot–Marie–Tooth 1A	Single-cell WGA followed by NGS	Direct diagnosis	1	NA	NA	No implantation data	Gui et al. [23]
SMA 1	Single-cell PCR or two-cell PCR	Direct diagnosis	3	NA	2/7		Fallon et al. [24]
SMA	Single-cell PCR	Direct diagnosis	5	6/62	6/9		Daniels et al. [25]
SMA	MARSALA	Direct diagnosis	2	NA	1/1		Ren et al. [26]
*Point mutation or small insertion/deletion*							
Charcot–Marie–Tooth X	Single-cell PCR or two-cell PCR	Direct diagnosis	1	1/12	1/2		Iacobelli et al. [27]
Charcot–Marie–Tooth X	Single-cell WGA followed by PCR	Linkage analysis	2	1/10	1/2		Borgulová et al. [28]
Charcot–Marie–Tooth 2F	Single-cell PCR or two-cell PCR	Direct diagnosis	1	2/11	2/4		Lee et al. [22]
Gerstmann-Straussler- Scheinker disease	Polar body PCR	Direct diagnosis	NA	2/14	2/2		Uflacker et al. [29]
FAP	Single-cell PCR	Direct diagnosis	10	3/93	3/25		Carvalho et al. [30]
FAP	Two-cell PCR	Direct diagnosis	1	2/10	2/3		Almeida et al. [31]

^a^ Direct diagnosis of the embryos without communicating results to the patients. NA = not available, SCA = spinocerebellar ataxia, SMA = spinal muscular atrophy, NGS = next generation sequencing, FAP = familial amyloidotic polyneuropathy.

**Table 3:** PGD of neurodegenerative diseases in our laboratory.

Case No.	Maternal Age	Disease	Inheritance	Gene	Mutation Type	Diagnostic Methodology	No. of Oocyte Retrieval Cycle	Embryos Diagnosed	Diagnostic Result (Unaffected/Affected)	No. of Embryo Transfer Cycle (No. of Embryos Transferred)	Pregnancy Outcome
1	30–34	SCA3	AD	*MJD1*	Trinucleotide expansion	Linkage analysis	1	6	2/4	1 (2)	No implantation
2	40–44	SCA3	AD	*MJD1*	Trinucleotide expansion	Linkage analysis	1	2	1/1	1 (1)	No implantation
3	25–29	SCA6	AD	*CACNA1A*	Trinucleotide expansion	Linkage analysis	2	12	6/6	2 (6)	No implantation
4	35–39	Charcot–Marie–Tooth 2E	AD	*NEFL*	Point mutation c.23C>G (p.P8R)	ARMS-qPCR + Linkage analysis	2	13	8/5	2 (8)	No implantation
5	25–29	Charcot–Marie–Tooth 2E	AD	*NEFL*	Point mutation c.23C>G (p.P8R)	ARMS-qPCR	1	7	5/2	2 (4)	Fraternal twins livebirth
6	30–34	Huntington’s disease	AD	*HTT*	Trinucleotide expansion	Linkage analysis	1	1	1/0	1 (1)	No implantation
7	30–34	ALS	AD	*FUS*	Point mutation c.1562G>A (p.R521H)	ARMS-qPCR + Linkage analysis	1	1	1/0	1 (1)	No implantation
8	35–39	FAP	AD	*TTR*	Point mutation c.349G>T (p.A117S )	ARMS-qPCR + Linkage analysis	1	2	0/2	0 (0)	No transfer
9 *	25–29	FAP	AD	*TTR*	Point mutation c.349G>T (p.A117S )	ARMS-qPCR	2	6	2/4	1 (2)	Identical twins livebirth
10	30–34	SMA	AR	*SMN1*, *SMN2*	Deletion in exon 7 of *SMN1*	ARMS-qPCR + Linkage analysis	1	9	3/6	1 (1)	Singleton livebirth

The mutations carried by the 10 patients are presumably inherited because all the patients have a familial history of neurodegenerative disease comparable with their clinical diagnosis. * indicates an index example with familial analysis and allele dropout we detailed in this paper. SCA = spinocerebellar atrophy, AD = autosomal dominant, *MJD1* = Machado–Joseph disease protein 1, *CACNA1A* = calcium voltage-gated channel subunit alpha1 A, *NEFL* = neurofilament light, ARMS-qPCR = amplification refractory mutation system quantitative polymerase chain reaction, *HTT* = Huntington, *ALS* = amyotrophic lateral sclerosis, *FUS* = FUS RNA binding protein, FAP = familial amyloidotic polyneuropathy, *TTR* = transthyretin, AR = autosomal recessive, *SMN1 =* survival of motor neuron 1, *SMN2* = survival of motor neuron 2.
